# Chronic Treatment with Metformin Has No Disrupting Effect on the Hepatic Circadian Clock in Mice

**DOI:** 10.3390/medicina58020293

**Published:** 2022-02-15

**Authors:** Nazmul Hasan, Koki Sugimoto, Koki Yamada, Jun-ichi Morishige, Kentaro Ushijima, Akio Fujimura, Naoto Nagata, Hitoshi Ando

**Affiliations:** 1 Department of Cellular and Molecular Function Analysis, Graduate School of Medical Sciences, Kanazawa University, Kanazawa 920-8640, Japan; hasannazmul32@gmail.com (N.H.); koko252521@stu.kanazawa-u.ac.jp (K.S.); kokiyamada@stu.kanazawa-u.ac.jp (K.Y.); morisige@med.kanazawa-u.ac.jp (J.-i.M.); 2 Division of Pharmaceutics, Faculty of Pharmaceutical Sciences, Sanyo-Onoda City University, Sanyo-Onoda 756-0884, Japan; k_ushijima@rs.socu.ac.jp; 3 Department of Pharmacology, School of Medicine, Jichi Medical University, Shimotsuke 329-0498, Japan; fujiakio@cc9.ne.jp

**Keywords:** metformin, circadian rhythm, clock genes, type 2 diabetes, liver

## Abstract

*Background and Objectives*: The antidiabetic agent metformin is known to activate AMP-activated protein kinase (AMPK) in various tissues. Because AMPK can modulate intracellular circadian clocks through regulating the stability of clock components, a single dose of metformin has been reported to affect circadian clocks in the peripheral tissues. In this study, therefore, we investigated whether chronic treatment with metformin causes the impairment of circadian clocks, especially if given at an inappropriate time. *Materials and Methods*: Non-diabetic C57BL/6J mice were allowed access to food only during 4 h at the beginning of the dark period, and repeatedly i.p. injected with a nearly maximum non-toxic dose of metformin, once daily either at 4 h after the beginning of the dark period or at the beginning of the light period. Diabetic ob/ob mice were given free access to food and treated with metformin in drinking water. Results: Under the controlled feeding regimen, 8-day treatment with metformin did not alter the mRNA expression rhythms of clock genes in both liver and adipose tissue of C57BL/6J mice, regardless of dosing time. In addition, chronic treatment with metformin for 2 weeks affected hepatic AMPK activation rhythm but did not disrupt the circadian clocks in the liver and adipose tissues of the ob/ob mice. *Conclusions*: These results mitigate concerns that treatment with metformin impairs peripheral circadian clocks, although confirmation is needed in humans.

## 1. Introduction

Various behavioral, physiological, and metabolic processes exhibit 24 h rhythmicity regulated by the intracellular circadian clocks [1]. In mammals, almost all types of cells possess this oscillator which is primarily composed of a transcription–translation-based autoregulatory feedback loop involving a set of several clock genes [2]. Specifically, the CLOCK-BMAL1 heterodimer activates the transcription of period (PER) and cryptochrome (CRY) genes, and the resultant PER–CRY complex inhibits the transcriptional activity of CLOCK-BMAL1, forming the negative feedback loop.

Recently, accumulating evidence suggests that circadian regulation is closely linked to metabolic homeostasis [3,4], and therefore that impairment of circadian clocks contributes to the development of metabolic diseases, including obesity and type 2 diabetes. For example, chronic jet lag (i.e., the systemic disruption of circadian clocks under an abnormal lighting condition) causes obesity in both mice [5] and shift workers [6]. The mutation of *Clock* induces obesity and metabolic syndrome in mice [7], and the haplotype of *CLOCK* is associated with a risk of obesity in humans [8]. In addition, experiments using tissue-specific *Bmal1* knockout mice revealed that the intracellular clocks in the liver [9], pancreas [10], skeletal muscle [11], and brown adipose tissue [12] play roles in regulating glucose production, insulin secretion, glucose uptake, and heat production, respectively. Furthermore, we found that circadian clocks are impaired in the peripheral tissues of both obese, diabetic mice [13,14] and patients with type 2 diabetes [15]. Thus, these results indicate the importance of maintaining circadian clock function in each tissue to prevent the development/progression of metabolic diseases.

Metformin is the most popular oral glucose-lowering medication, and its combination with lifestyle modifications is considered to be the optimal initial therapy for type 2 diabetes [16]. The molecular mechanisms of action of metformin are complex and remain incompletely understood in spite of its widespread use [17]. However, metformin is known to reduce hepatic glucose production partly via AMP-activated protein kinase (AMPK) activation [18]. AMPK is one of the major metabolic sensors and, interestingly, may transmit energy-dependent signals to circadian clocks [19]. Specifically, AMPK has been shown to destabilize CRY1 and to be required for circadian clock function in the liver of mice [20]. Moreover, Um et al. reported that AMPK promoted degradation of PER2 through the activation of casein kinase Iε, and therefore that a single dose of metformin led to a phase advance of circadian clocks in the peripheral tissues of mice [21]. These findings raise the possibility that treatment with metformin might cause impairment of circadian clocks, especially if given at an inappropriate time. To examine this hypothesis, we investigated the effect of chronic treatment with metformin on peripheral circadian clocks in mice.

## 2. Materials and Methods

### 2.1. Animals

Male C57BL/6J (*n* = 58) and ob/ob mice (*n* = 32) were purchased from Charles River Japan (Yokohama, Japan). All mice were maintained under controlled temperature and humidity with a 12:12-h light/dark cycle and fed a regular diet (CRF-1 (Oriental Yeast, Tokyo, Japan) for C57BL/6J mice; CE-2 (CLEA Japan, Tokyo, Japan) for ob/ob mice) and water ad libitum. All experiments were conducted after at least one week of acclimation.

### 2.2. Experiments

Metformin hydrochloride was obtained from FUJIFILM Wako Pure Chemical (Osaka, Japan). Zeitgeber time (ZT) was used to describe the experimental time with ZT 0 defined as lights on and ZT 12 as lights off. We carried out the three following experiments.

#### 2.2.1. Experiment 1: Effects of a Single Dose of Metformin in C57BL/6J Mice

Twelve-week-old C57BL/6J mice were divided into 4 groups (*n* = 4 per group) and intraperitoneally injected with 5 µL/g body weight of saline with or without metformin hydrochloride (150, 300, or 600 mg/kg body weight) at ZT 0. Chow was taken away and after 2 h (at ZT 2), blood glucose level in the blood (taken from the tail) was measured using a Glucocard G+ meter (Arkray, Kyoto, Japan), and the mice were euthanized to obtain liver samples.

#### 2.2.2. Experiment 2: Effects of Multiple Doses of Metformin in C57BL/6J Mice

**Figure 1:**
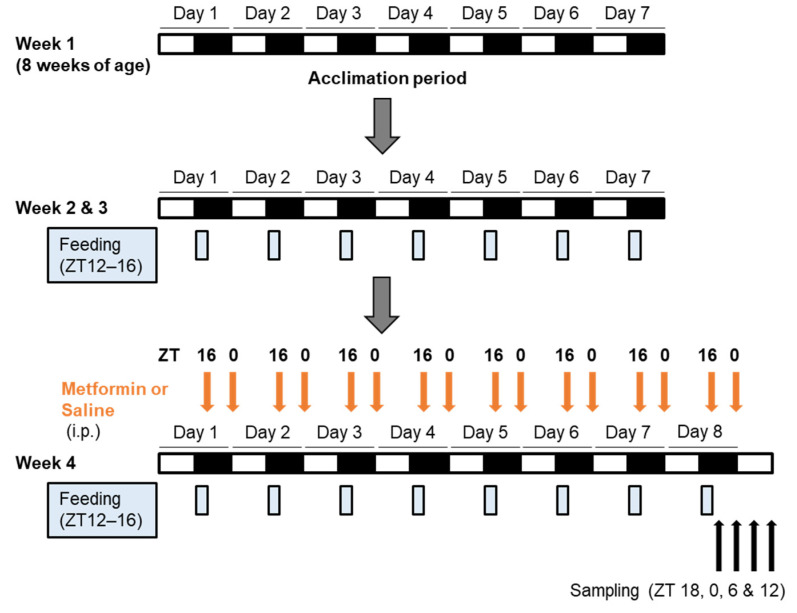
Protocol for the multiple-dose experiment.

As shown in Figure 1, C57BL/6J mice were obtained at 8 weeks of age, and allowed access to food only for 4 h during the active (dark) period (ZT 12 to ZT 16) from 9 weeks of age. At 11 weeks of age, mice were divided into 3 groups (*n* = 12–15 per group) and intraperitoneally administered saline with or without metformin hydrochloride (150 mg/kg body weight) twice daily at ZT 16 and ZT 0 for 8 days: control group (saline at both ZT 16 and ZT 0), ZT 16 group (metformin at ZT 16 and saline at ZT 0), and ZT 0 group (saline at ZT 16 and metformin at ZT 0). The dosing at ZT16 was conducted under dim red light. On the last day, the mice were euthanized to obtain to liver and epididymal fat samples at ZT 18, 0, 6, or 12.

#### 2.2.3. Experiment 3: Effects of Chronic Administration of Metformin in ob/ob Mice

Eight-week-old ob/ob mice were divided into 2 groups (*n* = 16 per group): the control group was provided with regular drinking water, whereas the treatment group was given 0.3% (*w*/*v*) metformin hydrochloride in drinking water. Both groups were fed chow ad libitum. After 2 weeks, non-fasting blood glucose level was measured and the mice were euthanized to obtain liver and epididymal fat samples at ZT 0, 6, 12, or 18.

### 2.3. Western Blotting Analysis

Frozen liver samples were homogenized in RIPA lysis buffer containing protease and phosphatase inhibitor cocktails (Nacalai Tesque, Kyoto, Japan) to prepare total protein lysates. Proteins (20 µg/lane) were separated by SDS-PAGE and then transferred to PVDF membranes. After overnight incubation with a primary antibody (AMPKα #2532 or phospho-AMPKα #2535, Cell Signaling Technology, Danvers, MA, USA) diluted 1:1000 at 4 °C, the membranes were incubated with an anti-rabbit IgG, HRP-linked antibody (#7074, Cell Signaling Technology) diluted 1:5000 for 1 h. The immunoreactive bands were imaged with ImageQuant LAS 4000 camera system (GE Healthcare, Chicago, IL, USA) after visualization with EzWestLumi plus (ATTO, Tokyo, Japan), and the pixel density was quantified with Image Studio Lite software ver 5.2 (LI-COR, Lincoln, NE, USA).

### 2.4. RNA Isolation and Real-Time Quantitative PCR

Total RNA was extracted from the frozen samples using an RNeasy Mini Kit or an RNeasy Lipid Tissue Mini Kit (Qiagen, Valencia, CA, USA), and cDNA was synthesized using a High-Capacity cDNA Reverse Transcription Kit (Thermo Fisher Scientific, Waltham, MA, USA). Gene expression was analyzed using real-time quantitative PCR with the Applied Biosystems ViiA 7 Real-Time PCR System (Thermo Fisher Scientific). Specific sets of primers and TaqMan probes (TaqMan Gene Expression Assays) were obtained from Thermo Fisher Scientific. The GenBank accession numbers, assay ID, and target exons, respectively, were: NM_007489.4, Mm00500222_m1, and 7–8 (*Bmal1*); Mm00478113_m1, NM_011066.3, and 19–20 (*Per2*); NM_007771.3, Mm00514392_m1, and 1–2 (*Cry1*); Mm00497539_m1, NM_016974.3, and 1–2 (*Dbp*); and NM_007475.5, Mm00725448_s1, and 7 (*Rplp0*). Data were analyzed using the comparative threshold cycle method with *Rplp0* as the internal control.

### 2.5. Statistical Analysis

Data are presented as the means and standard deviation (SD). Statistical differences were determined using Student’s *t*-test or one-way analysis of variance (ANOVA) followed by Dunnett’s post hoc test. The calculations were performed using IBM SPSS Statistics (version 24.0). A *p*-value <0.05 was considered to be significant.

## 3. Results

### 3.1. A High Dose of Metformin Activates AMPK with Toxicity

**Figure 2:**
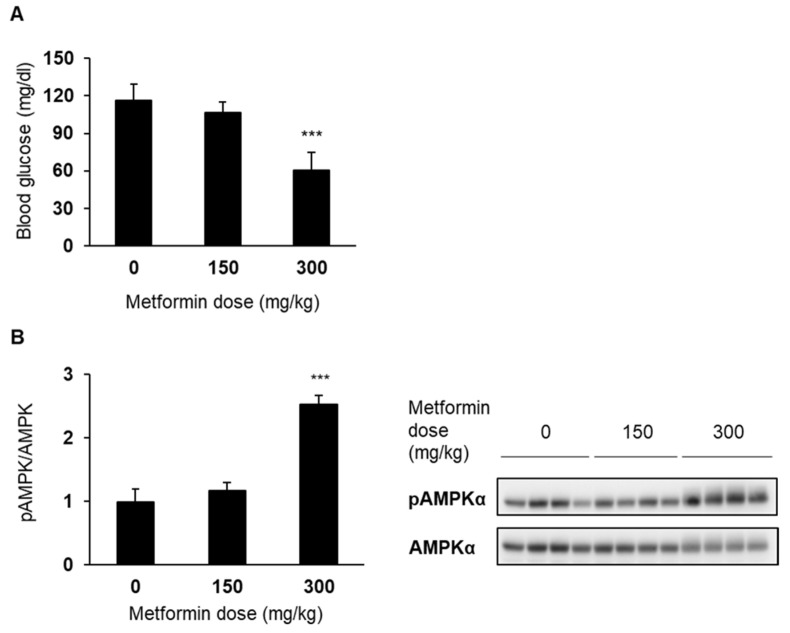
Effects of a single dose of metformin on blood glucose (**A**) and hepatic AMPK activation (**B**) in C57BL/6J mice. Blood glucose measurement and liver sampling were conducted at 2 h after administration of metformin. Data are presented as the mean + SD of 4 mice. *** *p* < 0.001 vs. vehicle group.

To identify an optimal dosage of metformin for the following experiments, we investigated the dose-dependent effect of its single dose on AMPK activation in non-diabetic C57BL/6J mice. The dosages (150, 300, and 600 mg/kg) were chosen based on the previous studies in which 250 mg/kg of metformin was administered to mice with [22] or without toxicity [23]. Within 30 min after dosing, reduced activity and piloerection appeared in the mice administered higher than or equal to 300 mg/kg of metformin. In addition, the toxic effects were unexpectedly severe, and all the mice receiving 600 mg/kg metformin died within 2 h. On the other hand, a dose of 150 mg/kg did not cause any obvious toxic effects. As shown in Figure 2A, 300 mg/kg, but not 150 mg/kg, of metformin reduced blood glucose level at 2 h after dosing. Consistent with these findings, the activating effect of metformin on hepatic AMPK was detected in the 300 mg/kg group, but not in the 150 mg/kg group (Figure 2B). In another preliminary experiment, some mice died after multiple once-daily doses of 200 mg/kg metformin (data not shown). Therefore, we chose the dosage of 150 mg/kg for the multiple-dose experiment.

### 3.2. Non-Toxic Repeated Doses of Metformin Have Minimal Effects on the Peripheral Circadian Clocks

**Figure 3:**
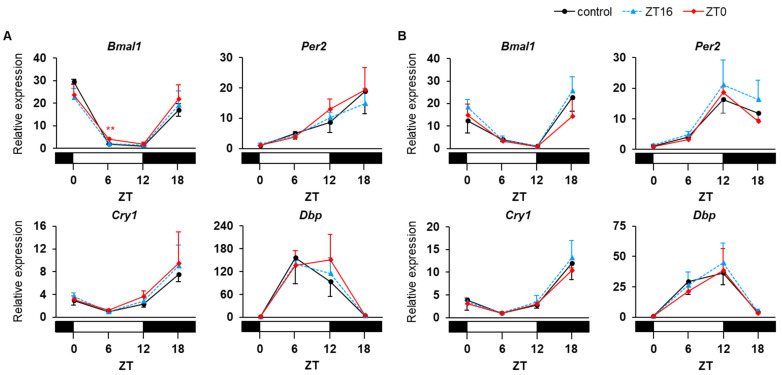
Effects of multiple doses of metformin on daily mRNA expression profiles of clock genes in the liver (**A**) and adipose tissue (**B**) of C57BL/6J mice. Mice were divided into 3 groups and administered saline with or without metformin twice daily for 8 days: control group (saline only, black circles and solid lines); ZT 16 group (metformin at ZT 16, blue triangles and dotted lines); and ZT 0 group (metformin at ZT 0, red diamonds and solid lines). Data are presented as the mean + SD of 3–4 mice per time point per group (*n* = 3, control group at each time point and the other groups at ZT 18). ** *p* < 0.01 vs. control group.

Metformin is known to impact appetite regulation possibly through both hypothalamic and gastrointestinal effects [24]. Most of peripheral circadian clocks, including those in the liver [25], adipose tissue [26], and uterus [27], are entrained strongly by food stimuli, rather than by light stimuli. Therefore, to investigate the direct effect of metformin on peripheral circadian clocks, we controlled food intake of mice using a 4 h time-restricted feeding regimen with food access at ZT 12–16 (equivalent to morning in humans) (Figure 1). In addition, to examine the effect of administration time of metformin, we selected two dosing times (ZT 16 and ZT 0, which are equivalent to the morning after breakfast and the evening after dinner in humans, respectively) and conducted injections of saline (with or without metformin) at both ZT 16 and ZT 0 to all of the mice. Under this strictly controlled condition, repeated administration of metformin did not obviously affect the mRNA expression rhythms of clock genes (*Bmal1*, *Per2*, *Cry1*, and *Dbp*) in the liver, regardless of dosing time (Figure 3A). Similarly, the treatment did not have significant impacts on the clock in the adipose tissue (Figure 3B).

### 3.3. Chronic Treatment with Metformin Has Minimal Effect on Hepatic Circadian Clock in Obese, Diabetic Mice

We next investigated the effects of chronic administration of metformin in ob/ob mice. This strain possesses a recessive mutation in the leptin gene, and consequently exhibits reduced locomotor activity, energy expenditure, and body temperature [28]. These mice also show obesity, insulin resistance, and hyperglycemia, and therefore are widely used as an obese, diabetic model. Moreover, circadian clocks in the liver and epididymal adipose tissue of this strain are significantly impaired [14], similar to circadian clock in the peripheral leucocytes of type 2 diabetic patients [15]. Therefore, we selected this model to investigate the effects of metformin in type 2 diabetic condition.

**Figure 4:**
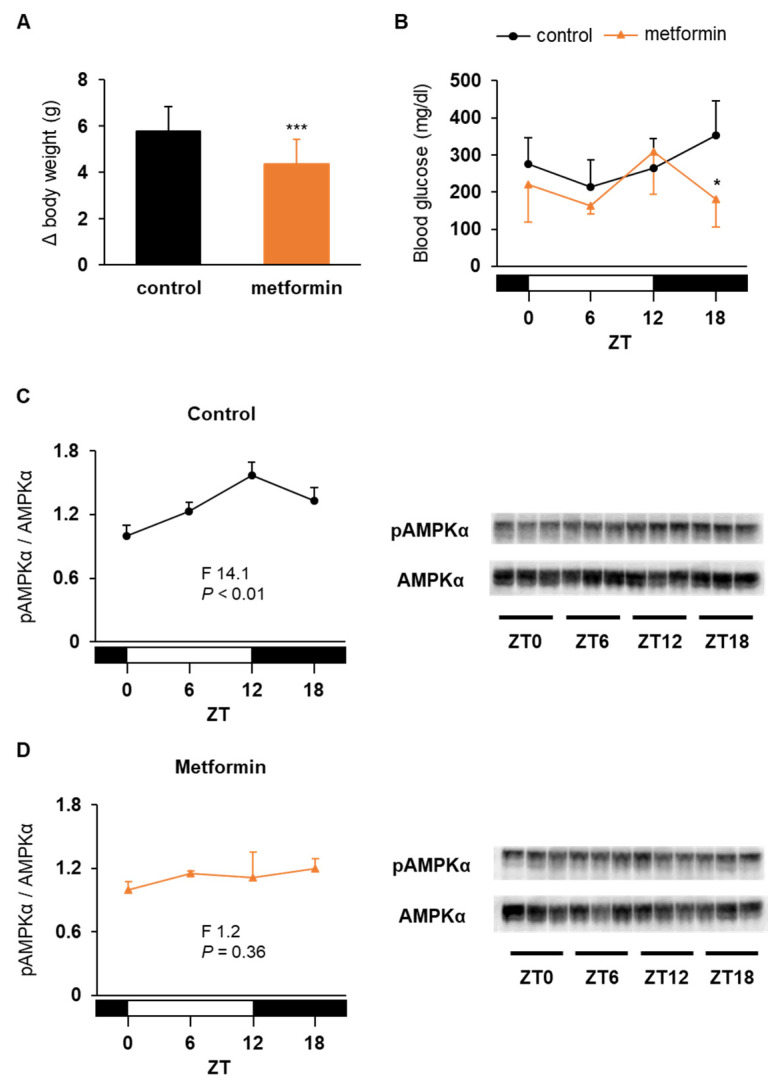
Effects of chronic administration of metformin on body weight gain (**A**), daily profiles of non-fasting blood glucose (**B**), and AMPK activation (**C**,**D**) in ob/ob mice. Mice were divided into 2 groups and given drinking water with (orange column, triangles, and lines) or without metformin (black column, circles, and lines) for 2 weeks. Data are presented as the mean and SD of 16 (**A**), 4 (**B**), or 3 mice (**C**,**D**). * *p* < 0.05, *** *p* < 0.001 vs. control group.

**Figure 5:**
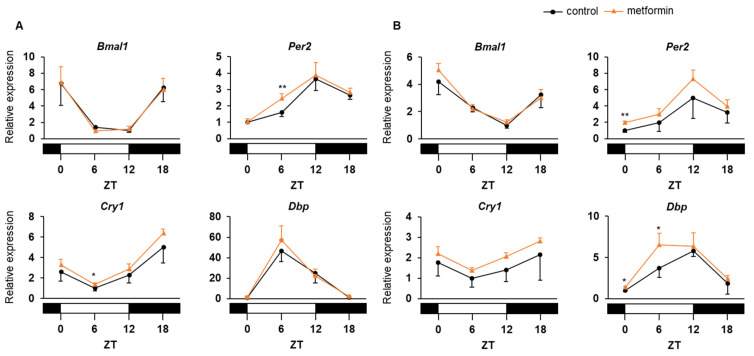
Effects of chronic administration of metformin on daily mRNA expression profiles of clock genes in the liver (**A**) and adipose tissue (**B**) of ob/ob mice. Mice were divided into 2 groups and given drinking water with (orange triangles and lines) or without metformin (black circles and lines) for 2 weeks. Data are presented as the mean and SD of 4 mice per time point per group. * *p* < 0.05, ** *p* < 0.01 vs. control group.

In this experiment, metformin was administered through drinking water, instead of once-daily administration, to increase daily dosage without toxicity. The dosage of metformin calculated from water intake was 389 ± 33 mg/kg/day, and drug-induced liver injury assessed by serum transaminase concentrations was not observed (aspartate and alanine aminotransferase 370 ± 114 and 431 ± 56 IU/L in the control group vs. 308 ± 83 and 353 ± 116 IU/L in the metformin group, respectively). Metformin treatment for 2 weeks significantly reduced water intake (8.1 ± 1.0 g/day in the control group vs. 6.5 ± 0.5 g/day in the metformin group; *p* < 0.05), food intake (5.7 ± 0.4 g/day in the control group vs. 5.1 ± 0.4 g/day in the metformin group; *p* < 0.01), and weight gain (Figure 4A). In addition, blood glucose level at ZT 18 was significantly lower in the treatment group than in the control group (Figure 4B). Consistent with this, daily rhythm of hepatic AMPK activation, which was observed in the control mice (Figure 4C), disappeared in the mice treated with metformin (Figure 4D). However, this chronic treatment did not profoundly alter the mRNA expression rhythms of clock genes in the liver although the differences of *Per2* and *Cry1* levels at ZT 6 were statistically significant (Figure 5A). In the adipose tissue, metformin increased *Dbp* mRNA level during the light period (Figure 5B).

## 4. Discussion

Orally administered metformin is rapidly absorbed in the proximal small intestine and immediately excreted unchanged in the urine [29] with a relatively short elimination half-life (3.8 h in humans [30] and 2.7 h in mice [31], respectively). These pharmacokinetic properties indicate the importance of dosing time for pharmacodynamics of metformin. Reportedly, both a single dose [21] and chronic administration [32] of metformin shift the phases of peripheral circadian clocks with the activation of AMPK in non-diabetic wild-type mice. Therefore, in this study, we investigated whether multiple doses of metformin also affect peripheral circadian clocks, especially depending on the dosing time. The present results reveal that repeated administration at a nearly maximum non-toxic dose has minimal effects on circadian clocks in the liver and adipose tissues of non-diabetic mice, regardless of dosing time.

Although it is certain that metformin activates AMPK, whether this drug exerts its glucose-lowering effect via AMPK remains questionable. For example, metformin can reduce both hepatic gluconeogenesis and blood glucose levels in mice lacking AMPK in the liver, and these effects are comparable to wild-type mice [33]. Moreover, metformin is thought to prevent mitochondrial ATP production and thus increase cytoplasmic AMP:ATP ratio, resulting in the inhibition of fructose-1,6-bisphosphatase, a key rate-controlling enzyme of gluconeogenesis [17]. In this study, when given to mice as a single dose, only a toxic dose of metformin obviously activated the hepatic AMPK, a modulator of intracellular circadian clock. Therefore, it seems reasonable that non-toxic repeated doses of metformin did not exert any effect on the peripheral circadian clocks. Further studies are needed to determine whether clinical doses of metformin influence circadian clocks in humans.

In the present study, chronic administration of metformin also had a minimal effect on the hepatic circadian clock in obese, diabetic mice, although the treatment affected hepatic AMPK activation. This result seems to be contradictory to a previous report that chronic treatment with metformin (164 mg/kg/day in the drinking water) advanced the phase of circadian clock in the liver of C57BL/6 mice [32]. However, these data were obtained under a constant dark condition, whereas our data were collected under a 12:12-h light/dark cycle. Because light is a strong zeitgeber (i.e., an external cue that entrains circadian clocks), the effect of metformin on hepatic clock might become obvious only in environments without zeitgebers.

We previously demonstrated that the oscillations of clock gene expression were substantially dampened in the liver and adipose tissues of the ob/ob mice, compared to the control mice [14]. In addition, acetylation levels of histone H3 at the promoter regions of clock genes were significantly lower in the adipose tissue of the ob/ob mice, and therefore treatment with a histone deacetylase inhibitor ameliorated the reduced expression of *Dbp* [34]. In the present study, chronic treatment with metformin for 2 weeks also improved the dampened expression of *Dbp* during the light period in the adipose tissue of the ob/ob mice. Similarly, Caton et al. reported that 7-day multiple doses (250 mg/kg/day, p.o.) of metformin increased clock gene expression in the adipose tissue of genetically obese, diabetic db/db mice [23]. Metformin increases acetylation of histones H3 and H4 via AMPK activation at least in vitro [35], suggesting one possible mechanism for its ameliorating effect on circadian clock in the adipose tissue.

## 5. Conclusions

In conclusion, repeated administration of metformin did not derange the mRNA expression rhythms of clock genes in the liver and adipose tissue of mice under a normal light condition, regardless of dosing time or dosing regimen. These results mitigate concerns that treatment with metformin impairs peripheral circadian clocks, although confirmation is needed in humans.

## Data Availability

The data presented in this study are available upon reasonable request to the corresponding author (H.A.).

## References

[1] Bass J., Takahashi J.S. (2010). Circadian integration of metabolism and energetics. Science.

[2] Takahashi J.S. (2017). Transcriptional architecture of the mammalian circadian clock. Nat. Rev. Genet..

[3] Green C.B., Takahashi J.S., Bass J. (2008). The Meter of Metabolism. Cell.

[4] Jan Stenvers D., Scheer F.A.J.L., Schrauwen P., la Fleur S.E., Kalsbeek A. (2018). Circadian clocks and insulin resistance. Nat. Rev. Endocrinol..

[5] Karatsoreos I.N., Bhagat S., Bloss E.B., Morrison J.H., McEwen B.S. (2011). Disruption of circadian clocks has ramifications for metabolism, brain, and behavior. Proc. Natl. Acad. Sci. USA.

[6] Pan A., Schernhammer E.S., Sun Q., Hu F.B. (2011). Rotating night shift work and risk of type 2 diabetes: Two prospective cohort studies in women. PLoS Med..

[7] Turek F.W., Joshu C., Kohsaka A., Lin E., Ivanova G., McDearmon E., Laposky A., Losee-Olson S., Easton A., Jensen D.R. (2005). Obesity and metabolic syndrome in circadian Clock mutant mice. Science.

[8] Sookoian S., Gemma C., Gianotti T.F., Burgueño A., Castaño G., Pirola C.J. (2008). Genetic variants of Clock transcription factor are associated with individual susceptibility to obesity. Am. J. Clin. Nutr..

[9] Ando H., Ushijima K., Shimba S., Fujimura A. (2016). Daily Fasting Blood Glucose Rhythm in Male Mice: A Role of the Circadian Clock in the Liver. Endocrinology.

[10] Perelis M., Marcheva B., Ramsey K.M., Schipma M.J., Hutchison A.L., Taguchi A., Peek C.B., Hong H., Huang W., Omura C. (2015). Pancreatic b cell enhancers regulate rhythmic transcription of genes controlling insulin secretion. Science.

[11] Harfmann B.D., Schroder E.A., Kachman M.T., Hodge B.A., Zhang X., Esser K.A. (2016). Muscle-specific loss of Bmal1 leads to disrupted tissue glucose metabolism and systemic glucose homeostasis. Skelet. Muscle.

[12] Hasan N., Nagata N., Morishige J.-I., Islam M.T., Jing Z., Harada K.-I., Mieda M., Ono M., Fujiwara H., Daikoku T. (2021). Brown adipocyte-specific knockout of Bmal1 causes mild but significant thermogenesis impairment in mice. Mol. Metab..

[13] Ando H., Yanagihara H., Hayashi Y., Obi Y., Tsuruoka S., Takamura T., Kaneko S., Fujimura A. (2005). Rhythmic Messenger Ribonucleic Acid Expression of Clock Genes and Adipocytokines in Mouse Visceral Adipose Tissue. Endocrinology.

[14] Ando H., Kumazaki M., Motosugi Y., Ushijima K., Maekawa T., Ishikawa E., Fujimura A. (2011). Impairment of peripheral circadian clocks precedes metabolic abnormalities in ob/ob mice. Endocrinology.

[15] Ando H., Takamura T., Matsuzawa-Nagata N., Shima K.R., Eto T., Misu H., Shiramoto M., Tsuru T., Irie S., Fujimura A. (2009). Clock gene expression in peripheral leucocytes of patients with type 2 diabetes. Diabetologia.

[16] (2021). American Diabetes Association Pharmacologic approaches to glycemic treatment: Standards of medical care in diabetesd2021. Diabetes Care.

[17] Rena G., Hardie D.G., Pearson E.R. (2017). The mechanisms of action of metformin. Diabetologia.

[18] Zhou G., Myers R., Li Y., Chen Y., Shen X., Fenyk-Melody J., Wu M., Ventre J., Doebber T., Fujii N. (2001). Role of AMP-activated protein kinase in mechanism of metformin action. J. Clin. Investig..

[19] Jordan S.D., Lamia K.A. (2013). AMPK at the crossroads of circadian clocks and metabolism. Mol. Cell. Endocrinol..

[20] Lamia K.A., Sachdeva U.M., Di Tacchio L., Williams E.C., Alvarez J.G., Egan D.F., Vasquez D.S., Juguilon H., Panda S., Shaw R.J. (2009). AMPK regulates the circadian clock by cryptochrome phosphorylation and degradation. Science.

[21] Jee H.U., Yang S., Yamazaki S., Kang H., Viollet B., Foretz M., Chung J.H. (2007). Activation of 5′-AMP-activated kinase with diabetes drug metformin induces casein kinase Iε (CKIε)-dependent degradation of clock protein mPer2. J. Biol. Chem..

[22] Henriksson E., Huber A.L., Soto E.K., Kriebs A., Vaughan M.E., Duglan D., Chan A.B., Papp S.J., Nguyen M., Afetian M.E. (2017). The Liver Circadian Clock Modulates Biochemical and Physiological Responses to Metformin. J. Biol. Rhythms.

[23] Caton P.W., Kieswich J., Yaqoob M.M., Holness M.J., Sugden M.C. (2011). Metformin opposes impaired AMPK and SIRT1 function and deleterious changes in core clock protein expression in white adipose tissue of genetically-obese db/db mice. Diabetes, Obes. Metab..

[24] Yerevanian A., Soukas A.A. (2019). Metformin: Mechanisms in Human Obesity and Weight Loss. Curr. Obes. Rep..

[25] Wu T., Jin Y., Ni Y., Zhang D., Kato H., Fu Z. (2008). Effects of light cues on re-entrainment of the food-dominated peripheral clocks in mammals. Gene.

[26] Ando H., Ushijima K., Fujimura A. (2013). Indirect effects of glucagon-like Peptide-1 receptor agonist exendin-4 on the peripheral circadian clocks in mice. PLoS ONE.

[27] Hosono T., Ono M., Daikoku T., Mieda M., Nomura S., Kagami K., Iizuka T., Nakata R., Fujiwara T., Fujiwara H. (2021). Time-Restricted Feeding Regulates Circadian Rhythm of Murine Uterine Clock. Curr. Dev. Nutr..

[28] Pelleymounter M.A., Cullen M.J., Baker M.B., Hecht R., Winters D., Boone T., Collins F. (1995). Effects of the obese gene product on body weight regulation in ob/ob mice. Science.

[29] He L. (2020). Metformin and Systemic Metabolism. Trends Pharmacol. Sci..

[30] Tucker G., Casey C., Phillips P., Connor H., Ward J., Woods H. (1981). Metformin kinetics in healthy subjects and in patients with diabetes mellitus. Br. J. Clin. Pharmacol..

[31] Junien J.L., Brohon J., Guillaume M., Sterne J. (1979). DBM mice as a pharmacological model of maturity onset diabetes: Studies with metformin. Arch. Int. Pharmacodyn. Ther..

[32] Barnea M., Haviv L., Gutman R., Chapnik N., Madar Z., Froy O. (2012). Metformin affects the circadian clock and metabolic rhythms in a tissue-specific manner. Biochim. Biophys. Acta—Mol. Basis Dis..

[33] Foretz M., Hébrard S., Leclerc J., Zarrinpashneh E., Soty M., Mithieux G., Sakamoto K., Andreelli F., Viollet B. (2010). Metformin inhibits hepatic gluconeogenesis in mice independently of the LKB1/AMPK pathway via a decrease in hepatic energy state. J. Clin. Investig..

[34] Ishikawa-Kobayashi E., Ushijima K., Ando H., Maekawa T., Takuma M., Furukawa Y., Fujimura A. (2012). Reduced Histone H3K9 Acetylation of Clock Genes and Abnormal Glucose Metabolism in ob/ob Mice. Chronobiol. Int..

[35] Galdieri L., Gatla H., Vancurova I., Vancura A. (2016). Activation of AMP-activated protein kinase by metformin induces protein acetylation in prostate and ovarian cancer cells. J. Biol. Chem..

